# Characterization of motility and piliation in pathogenic *Neisseria*

**DOI:** 10.1186/s12866-015-0424-6

**Published:** 2015-04-30

**Authors:** Jens Eriksson, Olaspers Sara Eriksson, Lisa Maudsdotter, Oskar Palm, Jakob Engman, Tim Sarkissian, Helena Aro, Mats Wallin, Ann-Beth Jonsson

**Affiliations:** Department of Molecular Biosciences, The Wenner-Gren Institute, Stockholm University, Svante Arrhenius väg 20C, SE-10691 Stockholm, Sweden; Theoretical Physics, KTH Royal Institute of Technology, Stockholm, Sweden

**Keywords:** *Neisseria*, Type IV pili, PilT, Twitching motility

## Abstract

**Background:**

The type IV pili (Tfp) of pathogenic *Neisseria* (i.e., *N. gonorrhoeae* and *N. meningitidis*) are essential for twitching motility. Tfp retraction, which is dependent on the ATPase PilT, generates the forces that move bacteria over surfaces. *Neisseria* motility has mainly been studied in *N. gonorrhoeae* whereas the motility of *N. meningitidis* has not yet been characterized.

**Results:**

In this work, we analyzed bacterial motility and monitored Tfp retraction using live-cell imaging of freely moving bacteria. We observed that *N. meningitidis* moved over surfaces at an approximate speed of 1.6 μm/s, whereas *N. gonorrhoeae* moved with a lower speed (1.0 μm/s). An alignment of the meningococcal and gonococcal *pilT* promoters revealed a conserved single base pair variation in the −10 promoter element that influence PilT expression. By tracking mutants with altered *pilT* expression or *pilE* sequence, we concluded that the difference in motility speed was independent of both. Live-cell imaging using total internal reflection fluorescence microscopy demonstrated that *N. gonorrhoeae* more often moved with fewer visible retracting filaments when compared to *N. meningitidis*. Correspondingly, meningococci also displayed a higher level of piliation in transmission electron microscopy. Nevertheless, motile gonococci that had the same number of filaments as *N. meningitidis* still moved with a lower speed.

**Conclusions:**

These data reveal differences in both speed and piliation between the pathogenic *Neisseria* species during twitching motility, suggesting a difference in Tfp-dynamics.

**Electronic supplementary material:**

The online version of this article (doi:10.1186/s12866-015-0424-6) contains supplementary material, which is available to authorized users.

## Background

The closely related pathogens *Neisseria gonorrhoeae* and *Neisseria meningitidis* colonize human mucosal epithelia, however, at different sites in the body. Neisserial motility is enabled by type IV pili (Tfp), which are long and dynamic filaments expressed by a phylogenetically diverse set of bacterial species, such as *Pseudomonas aeruginosa, Vibrio cholerae, Legionella pneumophila, Moraxella bovis, Escherichia coli* and *Myxococcus xanthus* (reviewed in [1]). In addition to mediating what is termed twitching motility, Tfp are also involved in a multitude of other functions, including attachment to host cells, microcolony and biofilm formation and DNA uptake [2-5]. Tfp can extend and retract via the assembly and disassembly of pilin subunits, called PilE, from an inner membrane protein pool [6,7]. The PilE of pathogenic *Neisseria* is divided into two classes: class I and class II pilin. The latter is only present in a subset of *N. meningitidis* strains. In contrast to class I pilin, class II pilin very rarely undergo antigenic variation and is shorter due to a deletion in the hypervariable region [8,9]. The assembled filaments can extend up to several micrometers from the bacterial surface in contrast to the bacterial diameter, which is approximately 1 μm. On the other hand, the pilus cross-sectional diameter is only 6–8 nm [10], far below the diffraction limit of visible light, which makes it impossible to observe single Tfp using bright field microscopy.

Tfp biogenesis and extension in *Neisseria* spp. depends on a core set of 12–15 highly conserved proteins [11-13], whereas the retraction of Tfp is powered by the ATPase PilT [14,15]. Based on X-ray crystallography data, the functional unit of PilT is proposed to be a hexamer [16,17]. In rod-shaped bacteria, such as *P. aeruginosa* and *M. xanthus*, pili are present at one pole although the ATPases necessary for assembly and retraction are distributed to both poles [18-21]. In pathogenic *Neisseria,* pili extend in all directions [22] and a study on PilT localization in *N. gonorrhoeae* indicated that it is found in the cytoplasm and associated with the inner membrane [14].

Of the two pathogenic *Neisseria* species, mainly *N. gonorrhoeae* has been used to study twitching motility. Optical tweezers experiments have demonstrated that a single gonococcal pilus is able to retract with peak forces up to 100 pN, and bundles of pili can exert even stronger forces [23,17]. The average pilus retraction rate in *N. gonorrhoeae* is 1.2 ± 0.2 μm/s at forces below 50 pN [24]. This rate may vary depending on multiple factors including the PilT concentration, the expression of the PilT paralog PilT2, the external force applied to the pilus and the oxygen concentration [25-28]. Movement of *N. gonorrhoeae* during long time scales is consistent with a random walk while persistent movement in one direction can be observed on shorter time scales (<15 s). Increasing the number of pili in *N. gonorrhoeae* increases the persistence time [29].

In this work, we have investigated motility in *N. meningitidis* and *N. gonorrhoeae*, including the influence of *pilE* sequence, pilin class and PilT expression. Since it is important to understand the relation between pilus filaments and bacterial motility, we also monitored the speed and the number of visible filaments during bacterial motility using a combination of phase contrast and total internal reflection fluorescence (TIRF) microscopy. We observe differences between gonococci and meningococci in their motility characteristics that are not related to PilT expression. *pilE* sequence or pilin class. Furthermore, we demonstrate that PilT expression is influenced by a species-specific single nucleotide variation in the *pilT* promoter.

## Results

### *N. gonorrhoeae* and *N. meningitidis* exhibit different speed during twitching motility

**Figure 1:**
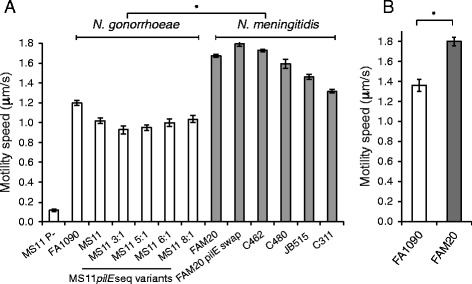
Average motility speed of *Neisseria* strains on a solid surface. **(A)** *N. gonorrhoeae* strains FA1090 and MS11, a nonpiliated MS11 and four clones of MS11 with different *pilE* sequences (i.e., 3:1, 5:1, 6:1, and 8:1). *N. meningitidis* strains FAM20 (serogroup C), C462 (serogroup W), C480 (serogroup C), JB515 (serogroup W), C311 (serogroup B) and the *pilE* sequence swap mutant in FAM20. The bacteria were allowed to adhere to poly-D-lysine-coated glass for 60 min at 37°C in 5% CO_2_-saturated GC broth. Attempts to track strains of serogroup A failed, as these strains did not adhere to the poly-D-lysine-coated surface. **(B)** Motility speed of *N. gonorrhoeae* FA1090 and *N. meningitidis* FAM20 on collagen-coated glass. The bacteria were allowed to adhere to the glass for 60 min prior to tracking. Motile bacteria were studied using live-cell imaging and particle tracking. The data are presented as the average values of at least 46 tracks acquired in at least three independent experiments. The error bars indicate the standard error of the mean. Significant differences (p < 0.05) between gonococcal and meningococcal strains are marked with an asterisk *.

To characterize motility in pathogenic *Neisseria*, we studied twitching motility using live-cell phase contrast imaging and automated particle tracking of moving bacteria. The bacteria were allowed to adhere to poly-D-lysine-coated glass and observed over a period of 60 s, with 12 images acquired per second. To determine the level of background noise in our system, a nonpiliated mutant derived from *N. gonorrhoeae* strain MS11 was included in the motility analysis and its speed was measured to 0.12 μm/s (Figure 1A). The tracking data revealed significant differences in the mean speed between the *N. gonorrhoeae* and *N. meningitidis* strains tested (Figure 1A). The average speed of *N. gonorrhoeae* strains MS11 and FA1090 ranged between 1.0-1.2 μm/s, whereas *N. meningitidis* strains had a speed of 1.4-1.7 μm/s which included strains of serogroup B (C311), C (C480 and FAM20) and W (C462 and JB515). PilE sequence variation in *Neisseria* affects the level of host cell adhesion and to determine whether it may also have an impact on motility, we tracked isogenic clones of MS11 with different *pilE* sequences [30]. Variants 3:1, 5:1 and 8:1 carry two amino acid substitutions in the variable mini-cassette (MC) 5 (a proline to serine and a serine to threonine substitution). Further, variants 3:1 and 8:1 both contain a lysine to glutamate substitution in MC6 and variant 8:1 has another five substitutions in MC4. MS11 6:1 has several amino acid changes in MC6, 5, 4 and 3, different from the other three MS11 *pilE* sequence variants. In addition, the variants 3:1, 5:1 and 6:1 have a larger substitution in the MC1 sequence. The hypervariable MC2 is unaltered in all *pilE* variants. Piliation level is similar between variants but approximately 50% lower in comparison to the parental MS11 strain [30]. Tracking analysis showed that there was no difference in speed between the PilE sequence variants and the parental MS11 strain (Figure 1A). In order to determine whether the PilE class affected motility speed, the FAM20 native *pilE* gene encoding a class II pilin was exchanged with the *pilE* sequence obtained from the *pilE* expression locus in the gonococcal strain FA1090, which is similar to class I pilins of *N. meningitidis*. Tracking analysis demonstrated that the PilE swap mutant of FAM20, although expressing slightly less *pilE* and pili (Additional file 1: Figure S1), moved with the same speed as the wild-type (Figure 1A), indicating that pilin of GC most likely is not in itself causing the lower speed of *N. gonorrhoeae*. The surface coating poly-D-lysine is positively charged at neutral pH. To confirm the difference in speed between meningococci and gonococci on a biologically relevant surface material with a neutral net charge at pH 7–8, we assessed twitching motility on collagen. As shown in Figure 1B, *N. gonorrhoeae* and *N. meningitidis* display a similar difference in speed on a collagen-coated surface. In conclusion, these data suggest that *N. meningitidis* moves faster than *N. gonorrhoeae* on both poly-D-lysine- and collagen-coated glass and that bacterial speed is independent of *pilE* sequence variation.

### Expression of *pilT* and *pilF* are equal but higher in *N. gonorrhoeae*

**Figure 2:**
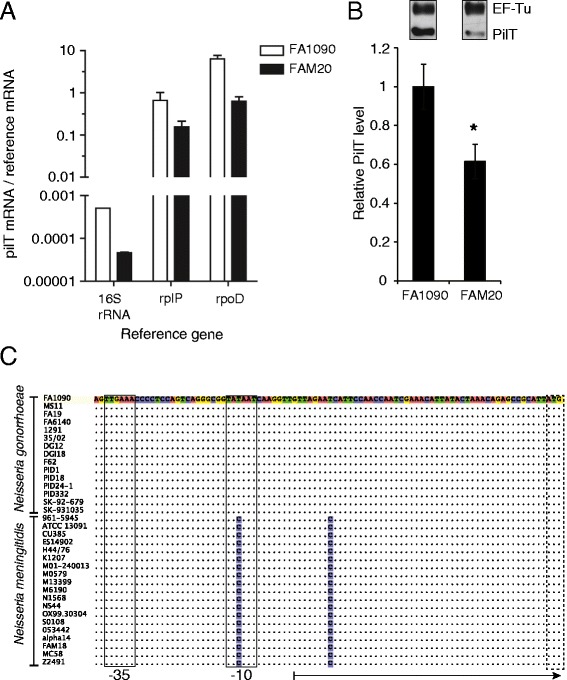
Analysis of PilT expression in *N. gonorrhoeae* strain FA1090 and *N. meningitidis* strain FAM20. **(A)** Bar chart showing average *pilT* mRNA levels in FA1090 and FAM20 normalized to 16S rRNA, 50S ribosomal gene *rplP* and σ factor *rpoD*. The error bars represent the standard deviation. The experiment was performed twice. **(B)** Representative immunoblots and PilT protein quantification. Bacterial lysates were separated on a 12% SDS-PAGE gel, probed with an anti-PilT antibody and an anti-EF-Tu antibody, and analyzed using an IR-scanner. EF-Tu migrates at 45 kDa, and PilT migrates at 37 kDa. The relative PilT/EF-Tu band intensities were averaged over more than three separate blots and normalized against FA1090. The error bars represent the standard error. Significant differences are marked with an asterisk * (p < 0.05 using an unpaired two-tailed Student’s *t*-test). **(C)** Alignment of the *pilT*-promoter region from sequenced gonococcal and meningococcal genomes. Dots indicate sequences identical to the reference FA1090 sequence, and sequence differences are highlighted. The boxes indicate the −35 and −10 promoter regions. The arrow denotes the main *pilT-*mRNA, and the dashed box indicates the *pilT* start codon.

The ATPases driving pili extension and retraction are PilF and PilT, respectively. PilT-mediated retraction is specifically essential for functional Tfp and twitching motility [7]. A previous study demonstrated a higher expression of *pilF* in gonococci in comparison to meningococci [31]. We could confirm higher levels of *pilF* mRNA in FA1090 than in FAM20 (Table 1). Because *pilF* expression differs between the species, we chose to also quantify PilT mRNA and protein levels by using quantitative real-time PCR and a two-color western blot assay with an in-house generated polyclonal PilT antibody raised against PilT from *N. gonorrhoeae*. Indeed, FA1090 expressed higher relative levels of *pilT* mRNA than FAM20, and *pilT* expression matched that of *pilF* in both species (Figure 2A and Table 1). The western blot, with elongation factor Tu (EF-Tu) as loading control, showed that *N. gonorrhoeae* FA1090 expressed nearly twice as much PilT protein as *N. meningitidis* FAM20 (Figure 2B). Because both the PilT and EF-Tu amino acid sequences of *N. gonorrhoeae* and *N. meningitidis* are identical, the antibodies should react equally well in both species. In conclusion, *N. gonorrhoeae* expresses more *pilF* and *pilT* in comparison to *N. meningitidis* and because the ATPase expression levels are matched it suggests that the PilT expression is not a factor underlying the difference in speed between pathogenic *Neisseria*.

**Table 1:** ***pilT*** **versus** ***pilF*** **mRNA expression**

**Strain**	**Reference gene**	***pilT*** **/reference gene**	***pilF*** **/reference gene**
FA1090	*16S rRNA*	5.04 E-04 (±2.12 E-06)	4.61 E-04 (±1.26 E-04)
	*rplP*	0.66 (±0.36)	0.53 (±0.12)
	*rpoD*	6.38 (±1.34)	5.46 (±0.71)
FAM20	*16S rRNA*	4.60 E-05 (±1.84 E-06)	4.12 E-05 (±1.41 E-06)
	*rplP*	0.16 (±0.06)	0.13 (±0.04)
	*rpoD*	0.64 (±0.17)	0.54 (±0.10)

### A single base pair variation alters *pilT* expression

**Figure 3:**
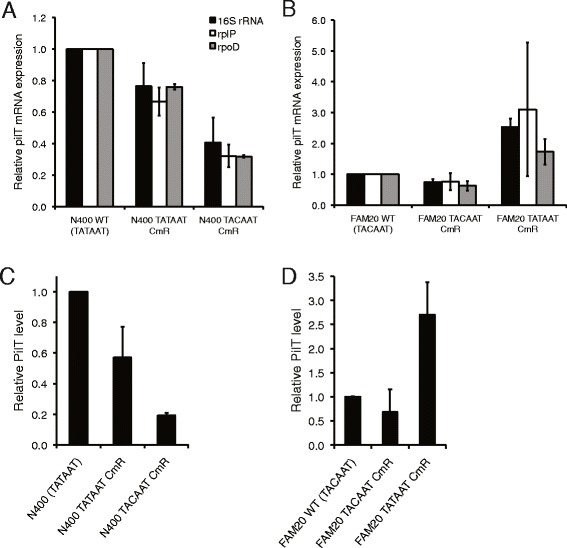
Analysis of PilT expression in *pilT* promoter mutants. The *pilT* promoter constructs used to transform *N. gonorrhoeae* N400 and *N. meningitidis* FAM20 carried either the 5’TATAAT or the 5’TACAAT sequence with a chloramphenicol resistance cassette (Cm^R^) oriented on the opposite strand and in the opposite direction of the *pilT* gene. *pilT* mRNA transcription level in N400 **(A)** and FAM20 **(B)**, as quantified using qPCR. mRNA expression was normalized to three different reference genes (i.e., 16S rRNA, 50S ribosomal protein *rplP* and σ factor *rpoD*) and compared to the WT level. The experiment was performed two to three times. The bars show the mean ± standard deviation. PilT protein quantification of whole cell lysates from N400 **(C)** and FAM20 **(D)** by western blot. The relative PilT/EF-Tu band intensities were averaged over two to four separate blots and normalized against WT strains. The error bars represent the standard deviation.

To determine the reason behind the different PilT expression in pathogenic *Neisseria*, we analyzed the *pilT* promoter. An alignment of the *pilT* promoter from representative strains is shown in Figure 2C and all available sequenced strains are shown in Additional file 2: Figure S2. While close to identical, the *pilT* promoter in a majority of *N. meningitidis* strains contained the −10 promoter element 5’TACAAT while the *pilT* promoter of all *N. gonorrhoeae* strains contained the canonical σ^70^ -10 sequence 5’TATAAT. An additional C/T polymorphism was found downstream of the transcriptional start site (Figure 2C). To experimentally investigate the effect of the -10 promoter sequence on PilT expression, we switched the *pilT* promoter between *N. meningitidis* and *N. gonorrhoeae*. We then assessed the role of the ATPase ratio in *Neisseria* motility. Promoter mutagenesis in the −10 sequence (see Material and Methods for detailed explanation of constructs and strains used) in both species demonstrated that the 5’TACAAT sequence confers lower *pilT* mRNA (Figure 3A, N400; Figure 3B, FAM20) and protein levels (Figure 3C, N400; Figure 3D, FAM20) without changing *pilF* mRNA expression (data not shown). Neither the motility speed or the level of piliation, quantified in the FAM20 mutants by using a whole cell ELISA and transmission electron microscopy (TEM), differed between the promoter variants (Additional file 3: Figure S3). In conclusion, the 5'TATAAT sequence specifically conserved in the gonococcal *pilT* promoter results in higher expression of PilT. However, the change in *pilT*/*pilF* ratio applied in this work does not significantly affect motility speed.

### Visualizing type IV pili in motile *N. gonorrhoeae* and *N. meningitidis*

**Figure 4:**
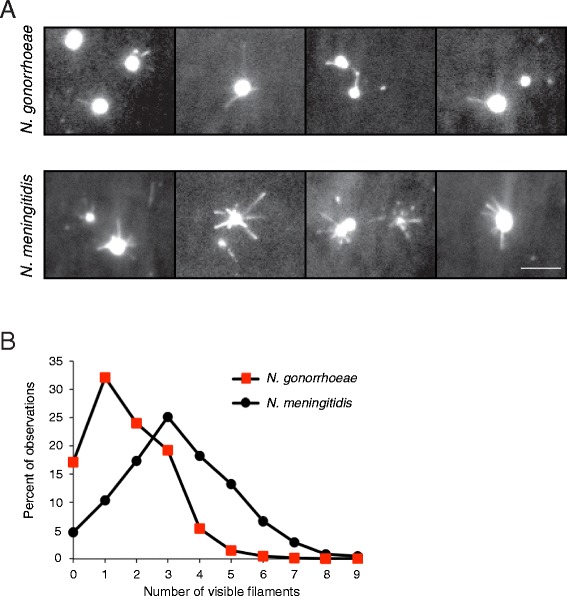
Visualization of Tfp during movement using TIRF microscopy. Live bacteria were stained with the Dylight™488 NHS-ester prior to experiments. **(A)** Representative pictures of stained *N. gonorrhoeae* and *N. meningitidis* crawling on a poly-D-lysine-coated surface. The scale bar corresponds to 5 μm. **(B)** Percent of observations with x number of visible filaments during movement on a solid surface. More than 2,600 individual frames from the time-lapse movies were analyzed for each species, and the number of visible filaments was counted in each frame.

**Figure 5:**
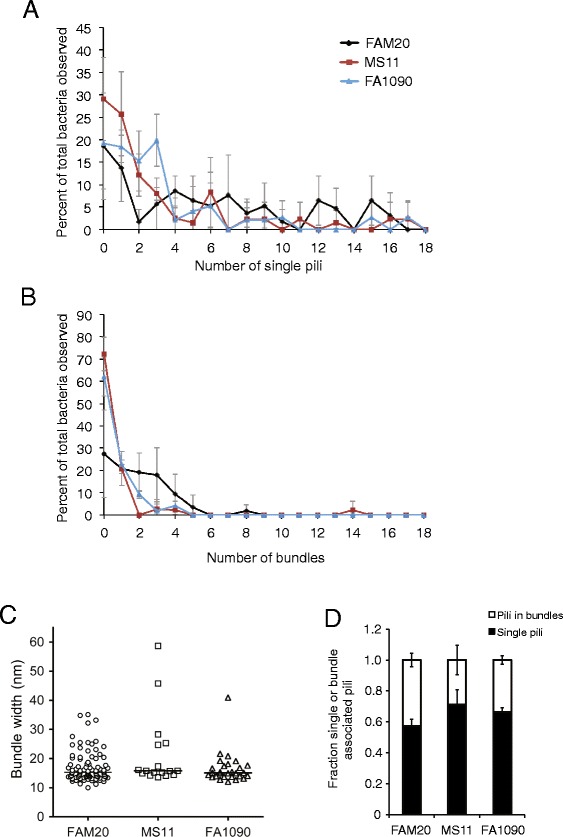
Quantification of piliation in FAM20, MS11 and FA1090 using transmission EM. **(A and B)** The graphs show the percentage of bacteria observed with x number of single pili **(A)** or pili bundles (irrespective of bundle width) **(B)** that appear to emanate from the bacteria. The total number of bacteria observed per strain were: FAM20 n = 48, MS11 n = 49, FA1090 n = 44. Significant differences was tested for by using *t*-test (two-tailed and non-equal variance), values are not significantly different. **(C)** Quantification of bundle width (nm), with each dot representing one bundle. The lines indicate the median bundle width. **(D)** Fraction of single and bundle-associated pili (from estimates of the number of pili in each bundle). The mean and standard deviation from two to three experiments are shown.

**Figure 6:**
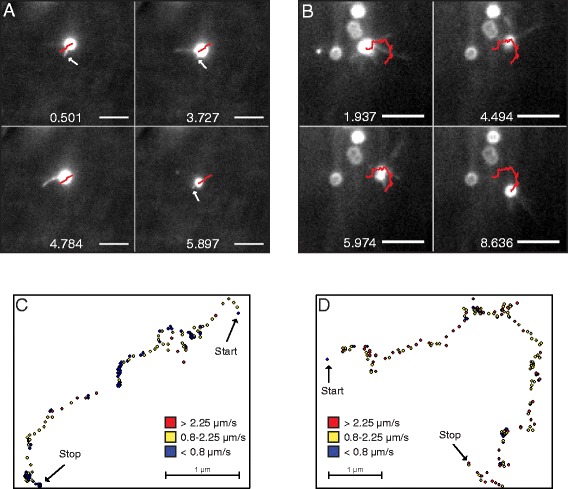
Analysis of Tfp-mediated motility using TIRF microscopy. Live bacteria were stained with the Dylight™488 NHS-ester and monitored using time-lapse microscopy. **(A)** Representative frames of *N. gonorrhoeae* strain FA1090 motility on a poly-D-lysine-coated glass surface. The arrows mark a short repetitively retracting filament. The scale bars correspond to 5 μm. **(B)** Representative frames of *N. meningitidis* strain FAM20 motility on a poly-D-lysine-coated glass surface. Several long filaments contribute to the movement of the bacterial cell. The red lines indicate the track of the bacteria. The numbers indicate the time in seconds. For videos, see supplemental Video S1 and Video S2. **(C and D)** The color-coded bacterial tracks were generated from Videos S1 (**C**; *N. gonorrhoeae*) and S2 (**D**; *N. meningitidis*). The colors indicate point velocities and are explained in the figure inset. The scale bars correspond to 1 μm.

To determine whether the observed difference in motility speed between *N. gonorrhoeae* and *N. meningitidis* could be linked to the distribution or number of pili, we stained bacteria and pili with an NHS-ester-based fluorescent dye and visualized pili using live-cell TIRF microscopy. The stain did not significantly affect speed (Additional file 4: Figure S4). TIRF microscopy provides a highly specific excitation of fluorophores up to ~100 nm from the glass surface. When using this illumination system, the background fluorescence is drastically reduced and the relatively weak fluorescence of stained pili becomes visible. Hence, it was possible to monitor pili in *Neisseria* during bacterial crawling, similar to previous visualization of Tfp in *Pseudomonas* [6]. The time for TIRF imaging was limited to 10–20 seconds due to fading of the fluorescent stain. It is important to note that this technique did not permit the distinction between a single pilus and a bundle of pili. Therefore, observed pili will collectively be referred to as visible filaments. We monitored bacterial crawling on a poly-D-lysine-coated glass surface and captured more than 100 bacterial tracks of *N. gonorrhoeae* FA1090 and MS11 and *N. meningitidis* FAM20 motility. In general, the number of visible filaments varied over time during the movement of a bacterium. Filaments were observed in all directions around the bacterium, which is in accordance with previous data [22]. Figure 4A shows representative images of motile, fluorescently stained *N. gonorrhoeae* and *N. meningitidis*. To quantify the relative frequency of visible filaments during motility, a manual single-blinded frame-by-frame counting was performed on the collected time-lapse movies (Figure 4B). When moving on a solid surface, *N. gonorrhoeae* most frequently displayed one filament, while *N. meningitidis* most often exhibited three filaments. Similar results as seen with TIRF were obtained after quantification of single pili and pili bundles in TEM (Figure 5A, single pili; 5B, pili bundles) although only a trend and not a significant difference could be detected. Comparison of pilus bundle size showed that the majority of bundles observed in both meningococci and gonococci contained two or three pili, corresponding to a bundle width of 10–25 nm (Figure 5C). The relative amount of pili that were present as single filaments versus as bundles was slightly higher in gonococcal strains than in FAM20 (Figure 5D). Figure 6A shows an example of Tfp-mediated motility involving primarily a single filament, as exemplified by *N. gonorrhoeae* strain FA1090 where the mean track speed was 0.97 μm/s and the distance travelled being 4.78 μm over the 5.4 s shown. The corresponding track of movement is presented in Figure 6C with dots indicating the position and the colors showing the speed in each frame. Figure 6B exemplifies bacterial movement by *N. meningitidis* strain FAM20 where the mean track speed was 1.46 μm/s and the distance travelled being 9.9 μm over the 6.7 s shown. The full time-lapse videos are shown in Video S1 and S2 (Additional file 5: Video S1; Additional file 6: Video S2). The track in Figure 6D indicates that meningococcal movement involving the apparent retraction of several filaments simultaneously results in more frequent changes in direction. Still, a graph relating the number of filaments to the motility speed only depicted a weak correlation and *N. gonorrhoeae* with the same number of filaments as meningococci in TIRF movies still moved at a lower speed (Additional file 7: Figure S5). To summarize, the results suggest that there is no correlation between the difference in number of filaments and motility speed in *N. meningitidis* and *N. gonorrhoeae*. Nevertheless, *N. meningitidis* FAM20 displayed multiple filaments more frequently than *N. gonorrhoeae*, which may influence changes in motility direction.

## Discussion

Tfp-mediated motility is accomplished via repeated steps of pilus assembly and PilT-mediated pilus retraction. In this work, we aimed to further characterize and quantify pilus dynamics and twitching motility in *N. meningitidis* and *N. gonorrhoeae*. Bacterial tracking demonstrated that the speed of *N. meningitidis* strains was significantly higher than that of *N. gonorrhoeae* strains (Figure 1). Live visualization of the filaments visible during twitching motility indicated that *N. meningitidis* FAM20 displays more pili than *N. gonorrhoeae* FA1090 and MS11 (Figure 4). However, the TIRF data did not reveal a clear correlation between the higher number of visible pili in *N. meningitidis* FAM20 and the higher motility speed (Additional file 7: Figure S5).

By using an NHS-ester-based fluorescent dye, we were able to observe Tfp during live neisserial motility. Newly synthesized unlabeled pili or pili bundles may have contributed to motility. However, we could also detect the elongation of labeled filaments (Additional file 6: Video S1), suggesting the possibility that labeled PilE subunits are recycled in *Neisseria*, analogous to previous reports in *P. aeruginosa* [6]. TIRF time-lapse experiments do not reveal whether a filament pulls, pushes, or is relaxed, but the observed straight shape of filaments is consistent with stretched filaments due to pulling. The reported narrow and solvent-inaccessible channel at the center as well as the flexibility of pili suggests a relatively small effect of Tfp pushing on motility [32]. The TIRF technique enables observation of labeled pili and bundles within 100 nm of the surface and filaments attaching at a large angle would be more difficult to image. However, given the fact that one pilus is 6–8 nm in diameter and the most frequent bundles are up to 24 nm in diameter, there is a large margin that should permit the observation of most filaments.

The presence of fewer visible filaments on moving *N. gonorrhoeae* in comparison to *N. meningitidis* suggests a difference in Tfp-dynamics between these two species. For *N. meningitidis,* the frames with the highest speed most often displayed several spread filaments, rather than several filaments pointing in the same direction (data not shown). The most clear trend from quantification of pili bundles in TEM indicate that *N. meningitidis* strain FAM20 more frequently display two to four bundles in comparison to *N. gonorrhoeae* (Figure 5B). Few published articles have explored the influence of pili bundles on motility. A recently published model suggested that persistence is largely dependent on the re-elongation of pili from stable Tfp membrane complexes and enhanced by small bundles containing two to three pili [22]. The formation of Tfp bundles is influenced by *pilE* sequence variation [33], increases with higher *pilE* expression [34] and is reduced in *N. gonorrhoeae* when grown in protein rich medium [35]. Bundles could in theory influence motility since they support stronger and more sustained retraction in comparison to single pili [35]. However, at this point our data does not clearly show a correlation between pili bundles and enhanced motility speed.

Meningococci express a polysaccharide capsule, which *N. gonorrhoeae* lacks. Unfortunately, an unencapsulated meningococcal mutant did not move on the tested surfaces, which made it impossible to compare this mutant with the wild-type strain. Quantification of the speed of serogroup A strains was not possible because these strains did not bind to the surface.

Analysis of *pilT* promoters and the mutagenesis experiment in pathogenic *Neisseria* strains revealed that a conserved difference in the σ^70^ -10 element between meningococci and gonococci (5’TACAAT and 5’TATAAT, respectively) is linked to a lower PilT expression in meningococci (Figures 2 and 3). The −10 promoter sequence 5’TACAAT has previously been linked to suboptimal transcription in *Helicobacter pylori* [36]. The mechanism behind the difference in *pilF* expression remains an open question despite a previous attempt to link expression levels to a repetitive DNA sequence present in the meningococcal but absent from the gonococcal *pilF* promoter [31]. The relative expression of PilT and PilF in pathogenic *Neisseria* are matched, suggesting that this balance is important for Tfp dynamics. Although the change in PilT expression and ATPase ratio through promoter mutagenesis reported here did not alter bacterial speed, a more considerable reduction in PilT expression has previously been demonstrated to influence Tfp dynamics in MS11 [26]. The amino acid sequence of PilT is identical between gonococci and meningococci, excluding intrinsic PilT differences as the source of motility variation. Because the PilT paralog PilT2 has been shown to increase pilus retraction in gonococci [28], we cannot exclude the possibility that differences in PilT2 expression between meningococci and gonococci might influence their relative speed. Optical tweezers experiments investigating *N. meningitidis* pilus retraction speed are lacking but the highly conserved Tfp biogenesis machinery and the similar pilus retraction force and speed in the more distantly related *M. xanthus* [25] support similar pilus retraction rates in both pathogenic *Neisseria* species. It is intriguing that all sequenced *N. meningitidis* strains have a suboptimal *pilT* promoter in comparison to *N. gonorrhoeae*. At this point, we can only speculate on the underlying reasons. One theory could be that events altering the expression of certain Tfp genes (e.g., *pilE* or *pilF*) may have disrupted the balance of Tfp biogenesis and function, driving the entire system back to homeostasis. Another hypothesis could be that the higher expression of ATPases is linked to optimal colonization of the gonococcal niche. The origin and further implications of this divergence remain to be investigated.

## Conclusion

In summary, due to a point variation in the *pilT −*10 promoter element, gonococci expressed a higher level of *pilT* in comparison to meningococci which is matched by a higher *pilF* expression. Furthermore, we observed distinct motility characteristics for *N. gonorrhoeae* and *N. meningitidis*, with respect to both the mean speed and the number of visible filaments during motility. However, the difference in motility speed between *N. gonorrhoeae* and *N. meningitidis* does not appear to be correlated with the difference in number of visible filaments or *pilE* sequence. Nevertheless, our data suggests a difference in Tfp-dynamics between these two species.

## Methods

### Bacterial strains, media and growth conditions

*N. gonorrhoeae* strains FA1090 (ATCC 700825), MS11 (ATCC BAA1833), N400 [37] and *N. meningitidis* strains FAM20 [38], C311, C480, C462 [39] and JB515 [38] were grown at 37°C in 5% CO_2_ on GC agar plates (GC medium base; Acumedia, Neogen Corporation, Lansing, MI, USA). The PilE sequence variants 3:1, 5:1, 6:1, and 8:1 of MS11 [40,30] and the PilT-deficient mutant of FAM20 [41] were described previously. *E. coli* strain DH5α was used for cloning and plasmid propagation, and strain BL21 (DE3) was used for protein expression. *E. coli* strains were maintained on LB agar plates (Acumedia). Liquid cultures of *Neisseria* were propagated in GC broth supplemented with Kellogg’s supplement. Liquid cultures of *E. coli* were grown in LB broth (Acumedia). Antibiotics were used at the following concentrations: 100 μg/ml for ampicillin and 50–100 μg/ml for kanamycin.

### Generation of mutants in *N. gonorrhoeae* and *N. meningitidis*

Two synthetic *pilT* promoter constructs were designed based on the FAM18 *pilT* upstream sequence, the *pilT* promoter and the *pilT* gene [GenBank NMC0036]. The only difference between these constructs was a single nucleotide in the σ^70^ -10 TATA box promoter element (5’TATAAT or 5’TACAAT). A chloramphenicol resistance cassette was placed upstream and in the opposite direction of the *pilT* promoter. The constructs were ordered from DNA2.0 (Menlo Park, CA, USA) and the sequences of the constructs are available from the authors on request. FAM20 and N400 were transformed with the constructs, which replaced the endogenous *pilT* region. Clones were selected on chloramphenicol plates (5 μg/ml). The MS11 derivate N400 was used due to its inducible expression of *recA6*, which renders this strain deficient in homologous recombination and *pilE* antigenic variation when grown in the absence of the inducer IPTG. A *pilE* sequence swap mutant in FAM20 was made by replacing the native *pilE* sequence with a construct containing the *pilE* expression locus from FA1090 under the control of the native FAM20 *pilE* promoter. A kanamycin resistance cassette inserted downstream of the FA1090 *pilE* gene enabled selection. Correct insertion and sequence was confirmed via sequencing of the mutant strains.

### Generation of PilT-specific antibodies

The *pilT* gene was PCR amplified from *N. gonorrhoeae* strain MS11 genomic DNA using the primers PilTfw AAGCTTCATATGCAGATTACCGACTTACTC and PilTrev CTTAAGCTCGAGGAAACTCATACTTTCGCTGTTT and cloned into the *NdeI-XhoI* sites of pET21-b to generate pET21-PilT. The insert was sequenced, and the plasmid was transformed into *E. coli* strain BL21(DE3) for protein expression and purification. The polyhistidine-tagged PilT was purified using Talon® resin and a combined batch/gravity flow protocol, according to the manufacturer’s instructions (Clontech Laboratories, Inc., Mountain View, CA, USA). Protein fractions of >95% purity, as determined using sodium dodecyl sulfate polyacrylamide gel electrophoresis (SDS-PAGE), were used to immunize rabbits. Rabbit immunizations were performed according to the institutional guidelines and approved by an ethical committee. All protocols were approved by the Swedish Ethical Committee on Animal Experiments (Approval ID: C93/08). IgG antibodies were purified from rabbit sera using Dynabeads® Protein G, according to the manufacturer’s instructions (Invitrogen). Highly specific polyclonal anti-PilT IgG antibodies were obtained by absorbing purified IgG antibodies against a whole-bacteria lysate from PilT-deficient *N. gonorrhoeae*.

### Quantitative real-time PCR analysis

The bacteria were grown on GC plates for 18 hours. Total RNA was isolated using a modified protocol for the SV Total RNA Purification kit (Promega, Madison, WI, USA), including an initial phenol/ethanol incubation on ice to stabilize the RNA and prevent degradation. RNA yield and quality were assessed using a NanoDrop 8000 (Thermo Fisher Scientific, Waltham, MA, USA). A 150 ng RNA sample from each strain was reverse-transcribed into cDNA with random hexamers using Superscript III First-Strand Synthesis (Invitrogen, Carlsbad, CA, USA). Quantitative real time PCR was performed using the LightCycler® 480SYBR Green I Master kit (Roche Diagnostics, Basel, Switzerland) with primer pairs (Eurofins MWG Operon (Ebersberg, Germany); listed in Table 2) in a LightCycler® 480 Real-Time PCR System (Roche Diagnostics). The primers were used at a final concentration of 250 nM (for *pilT, pilF* and *pilE* swap), 400 nM (for 16S rRNA) and 500 nM (for *rplP*, *rpoD* and *pilE*). The qPCR program was adapted from the LightCycler® 480SYBR Green I Master kit (Roche Diagnostics), with an annealing temperature of 62°C. Data analysis was performed with the LightCycler® 480 Software 1.5 using the comparative cycle threshold method, in which the target mRNA is normalized to the reference genes. The primer specificity was controlled using melting curve analysis. The experiment was performed at least twice.

**Table 2:** **List of qPCR primer pairs**

**Gene**	**Primer sequence 5’-3’**	**Reference**
*pilT* fwd	GTCGACCGTATCGTGGACGTATT	[43]
*pilT* rev	TTCAGCAGGTTTTGGGAGATGAC	[43]
*16S rRNA* fwd	TTTGATCCTGGCTCAGATTG	this work
*16S rRNA* rev	TATGTTACTCACCCGTTCGC	this work
*rplP* fwd	GTGGCGGTAAAGGTAACGTGGAAT	[42]
*rplP* rev	TCGAATGCTTCACGAGCCAGTT	[42]
*rpoD* fwd	CCTGACGATTGAGGAACAAC	this work
*rpoD* rev	TCGATTTGATCGGCATCGGA	this work
*pilF* fwd	CGCTGCTTTGAAGTCTTTCC	this work
*pilF* rev	CACCATATGCCCTGTTTGTGC	this work
*pilE* fwd	TATTCCGACAACGGCACATTCCC	[42]
*pilE* rev	CCTTCAACCTTAACCGATGCCA	[42]
*pilE swap* fwd	ATCGCTATCGTCGGCATTTT	this work
*pilE swap* rev	CGGCTGATTTTTGACCTTCG	this work

### Western blotting

Bacteria were harvested from GC agar plates in PBS to achieve an OD_600_ = 1. The bacterial suspensions were normalized according to the total protein content, as determined using a Bradford total protein assay (Bio-Rad, Hercules, CI, USA), and equal amounts of total protein were separated using 12% SDS-PAGE. All samples were boiled in reducing sample buffer at 95°C for 5 min prior to electrophoresis. The proteins were transferred from the gel onto PVDF sheets and overlaid with primary antibodies: rabbit anti-PilT (1:10,000) and mouse anti-EF-Tu (1:2,000). Incubation with the primary antibodies was followed by two different fluorescent dye-conjugated secondary antibodies for the detection of PilT (goat anti rabbit IgG IRdye800CW (Li-COR)) (1:10,000) and for the detection of EF-Tu (goat anti-mouse IgG IRdye680 (Li-COR)) (1:20,000). The membrane was visualized and analyzed using an Odyssey IR scanner (Li-COR, Lincoln, NE, USA) at 700 and 800 nm. See the Figure legends for information concerning the number of repeats.

### ELISA

The ELISA was performed as described previously [42]. Briefly, the wells of a 96-well microtiter plate were coated with 50 μl of either a bacterial suspension in PBS (OD_600_ = 0.005) or diluted whole bacterial protein extracts. To prepare whole bacterial protein extracts, one ml of a bacterial suspension at OD_600_ = 0.32 in PBS was mixed with 100 μl of trichloroacetic acid, followed by a 15 min incubation on ice. After a 5 min centrifugation at 20,000× g, the pellet was dissolved in PBS and diluted before being loaded into a 96-well plate. Equal loading was determined using a Bradford total protein assay (Bio-Rad). The samples were allowed to adhere for 2 h and blocked with 5% bovine serum albumin (BSA) for 2 h at room temperature. The samples were incubated for 1 h at 37°C and 5% CO_2_ with an anti-FAM20 pilus antibody (1:5,000) [38] and subsequently incubated with an HRP-conjugated anti-rabbit antibody (1:5,000) for another hour at 37°C. HRP was detected using 3, 3’, 5, 5’-tetramethylbenzidine (TMB), and the reaction was stopped using 1 M HCl. The absorbance at OD_450_ was read using a microplate reader. The experiment was performed twice in triplicate.

### Transmission electron microscopy

Bacteria that were grown for 16–18 h on GC plates were allowed to settle onto formvar- and carbon-coated EM grids (Carbon Type-B on 200 mesh Formvar-coated Copper grid, Caspilor, Sweden) for 40–60 min in GC broth with 10% Kellogg’s supplement at 37°C/5% CO_2_. The bacteria were subsequently fixed in 1% glutaraldehyde in 0.1 M phosphate buffer, washed and stained with 1% uranyl acetate. The sections were examined with a Tecnai G2Spirit BioTWIN microscope (FEI Company, Eindhoven, The Netherlands) at 80 kV. Digital images were obtained using a Gatan US1000 CCD camera (Gatan Inc., Pleasanton, CA, USA) at a magnification of 18,500. Pili were quantified manually using Image J software. The bundle size was approximated using the Image J software. The experiment was performed two to three times. Single-blinded picture acquisition and pilus quantification was performed.

### Amino-labeling of bacteria for TIRF microscopy

A 1 μl loop of bacteria was carefully resuspended in 60 μl of sterile PBS (corresponding to an OD_600_ ≈ 5), to which 3 μl of a DyeLight™ 488 NHS Ester (Thermo Scientific, Thermo Fisher Scientific) suspension was added (10 mg/ml, diluted in dimethylformamide). After a 10 min incubation at 37°C, 5 μl of the labeled bacterial suspension was added to 35 mm poly-D-lysine-coated glass bottom dishes (MatTek®, Ashland, MA, USA) containing 3 ml of GC broth supplemented with 10% Kellogg’s supplement and pre-warmed to 37°C. The labeled bacteria were allowed to incubate for 20 min at 37°C in a 5% CO_2_ atmosphere in the microscope prior to the start of the experiment. Live-cell time-lapse analysis was performed with TIRF microscopy using a connected 488 nm argon laser and a 100x objective (N/A1.46, Carl Zeiss, Oberkochen, Germany). Images were captured using an EM-CCD camera (Hamamatsu, Hamamatsu City, Japan). A minimum of 2,600 frames, representing a total of approx. 150 s, was captured for each strain from at least two separate occasions.

### Live-cell imaging, tracking, and data analysis

Half a 1 μl loop of bacteria that were grown on GC agar plates for 16–18 h were gently suspended in 200 μl of GC broth. Then, 10 μl of the bacterial suspension was added to 3 ml of pre-warmed GC broth containing 10% Kellogg’s supplement in 35 mm poly-D-lysine-coated or collagen-coated glass bottom dishes (MatTek®) and allowed to incubate for 1 h prior to microscopy. The cell culture dishes were transferred to a humidified incubation chamber (37°C, 5% CO_2_) connected to an inverted fluorescence microscope (Cell observer Z1, Carl Zeiss). The images captured during the time-lapse experiments were further processed using Axiovision® software (Carl Zeiss) and ImageJ (NIH, Bethesda, MD, USA). The tracking of bacteria was performed using the automatic tracking module in the Axiovision® software suite, version 4.7, and each individual track was manually inspected for automatic tracking errors. Filament localization and counting were performed manually in ImageJ on a frame-by-frame basis, using the Point-picker tool. Time-lapse movies were analyzed in a single-blinded manner.

### Motility tracking observation criteria

A total of 743 phase contrast images were acquired for a duration of 60 s per track on each of two to three independent experimental days. Bacteria were tracked if their positions differed by at least 2 μm between T_o_ and T_60_ and if they stayed adhered to the glass surface for the entire image acquisition period. Bacteria that moved within 10 μm of each other or debris in the media were not tracked. The fields of view were selected for the presence of motile bacteria at a low bacterial density.

### Statistical methods

The mean motilities between tracks and PilT expression levels were compared using unpaired *t*-tests. Differences between multiple groups were analyzed using analysis of variance (ANOVA) followed by Tukey’s honestly significantly different (HSD) post-hoc test. Statistical differences between ratios were analyzed after log transformation of the data.

## Additional files

Additional file 1: Figure S1. Expression of *pilE* mRNA and piliation in the FAM20 *pilE* sequence swap mutant. **(A)** *PilE* mRNA expression was normalized to the three reference genes (i.e., 16S rRNA, 50S ribosomal protein *rplP* and σ factor *rpoD*) and compared to the WT level. The experiment was performed two times. The bars show the mean ± standard deviation. **(B-C)** The graphs show the percentage of bacteria observed with x number of single pili **(B)** or pili bundles **(C)** that appear to emanate from the bacteria. The total number of bacteria observed per strain were: FAM20 WT n = 10 and FAM20 pilE swap n = 9. Additional file 2: Figure S2. Alignment of the *pilT*-promoter region from all sequenced gonococcal and meningococcal genomes. Additional file 3: Figure S3.
*pilT* promoter mutants in N400 **(A)** and FAM20 **(B)** were observed using live-cell microscopy, and tracks were analyzed by particle tracking. The data are presented as the average values of at least 40 tracks acquired in two to three independent experiments. The error bars indicate the standard error. The level of surface-exposed pili on meningococcal strains was quantified with whole cell ELISA **(C)** using an anti-pili antibody that primarily recognizes PilE. The bar chart shows the relative surface-exposed pili levels of the bacterial strains. The absorbance value of FAM20 WT was set to 1.0. The FAM20 ∆*pilT* strain was included as a hyperpiliated control. The bars represent the mean ± standard deviation from two separate experiments. Quantification of piliation in FAM20 *pilT* promoter mutants using TEM **(D and E)** The graphs show the percentage of bacteria observed with x number of single pili **(D)** or pili bundles (irrespective of bundle width) **(E)** that appear to emanate from the bacteria. The total number of bacteria observed per strain were: TATAAT CmR n = 41, TACAAT CmR n = 26. Results from FAM20 WT in Figure 5 are included as reference. The mean from two to three independent experiments are shown. Additional file 4: Figure S4. Average motility of *Neisseria* strains stained with NHS-ester-based fluorescent dye and observed using live-cell TIRF microscopy. The average speed for gonococcal strains was 0.9 ± 0.4 μm/s (N = 19 tracks), and the average speed for meningococcal strains was 1.7 ± 0.4 μm/s (N = 11 tracks). ± denotes the standard deviation. Additional file 5: Video S1. Movie showing an example of the Tfp-mediated motility that is often observed in *N. gonorrhoeae* strain FA1090. The bacteria were labeled with the DyLight™ 488 NHS-ester and visualized using TIRF illumination at 505 nm excitation and 514 nm emission wavelengths. Motion takes place via the retraction of a small number of filaments. The time in seconds is shown at the left. The scale bar is 5 μm. Additional file 6: Video S2. Tfp-mediated motility in *N. meningitidis* strain FAM20 involving several filaments. The bacteria were labeled with the DyLight™ 488 NHS-ester and visualized using TIRF illumination at 505 nm excitation and 514 nm emission wavelengths. The video shows a U-shaped pathway with occasional pausing. Several long filaments are visible. The time in seconds is shown at the left. The scale bar is 5 μm. Additional file 7: Figure S5. Relation between the average motility speed and the number of visible filaments in bacteria observed by TIRF. A frame-by-frame analysis of active filaments and bacterial speed in more than 2600 frames for each species was performed. The error bar corresponds to the standard deviation of the velocity.

## Abbreviations

EF-Tu: Elongation factor Tu
iEM: Immunogold electron microscopy
TEM: Transmission electron microscopy
TIRF: Total internal reflection fluorescence
Tfp: Type IV pili
WT: Wild-type
