# Supplementary figures and images for: Characterization of motility and piliation in pathogenic Neisseria
Source: BMC Microbiol. 2015 Apr 30;15:92. doi: 10.1186/s12866-015-0424-6 (PMC4449605; doi:10.1186/s12866-015-0424-6)

# Figure S1

**A**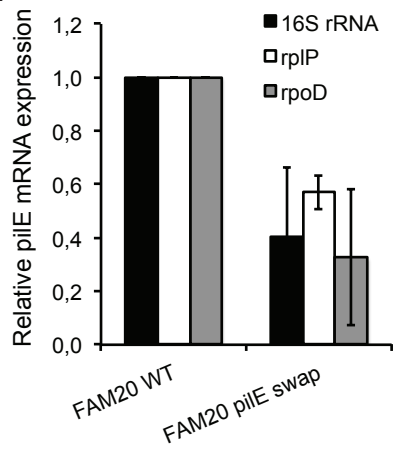**B**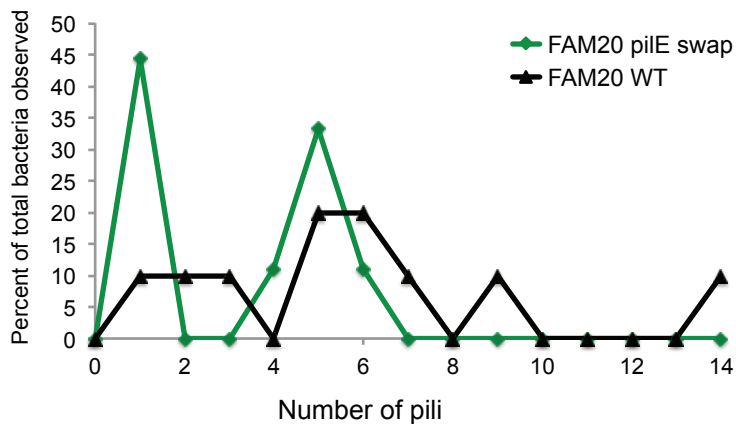**C**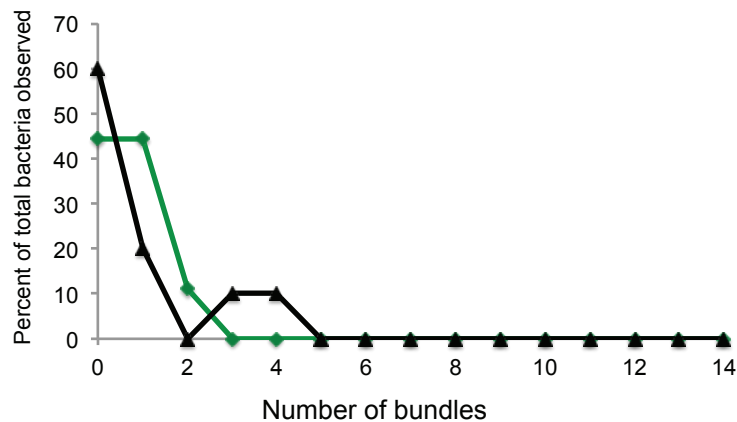

Supplement: Additional file 1: Figure S1. — Expression of pilE mRNA and piliation in the FAM20 pilE sequence swap mutant. (A) PilE mRNA expression was normalized to the three reference genes (i.e., 16S rRNA, 50S ribosomal protein rplP and σ factor rpoD) and compared to the WT level. The experiment was performed two times. The bars show the mean ± standard deviation. (B-C) The graphs show the percentage of bacteria observed with x number of single pili (B) or pili bundles (C) that appear to emanate from the bacteria. The total number of bacteria observed per strain were: FAM20 WT n = 10 and FAM20 pilE swap n = 9. [file 12866_2015_424_MOESM1_ESM.pdf]

# Figure S3

**A**

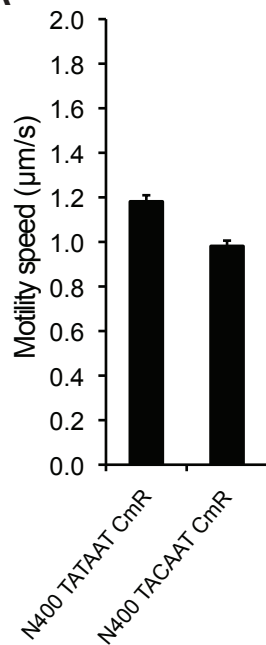

**B**

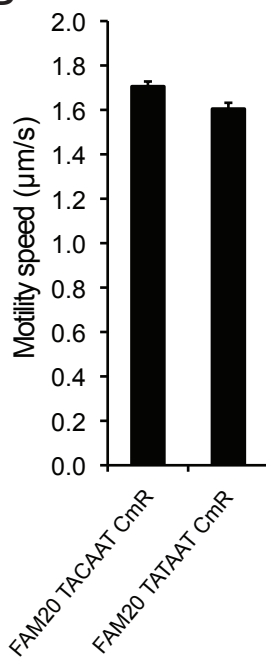

**C**

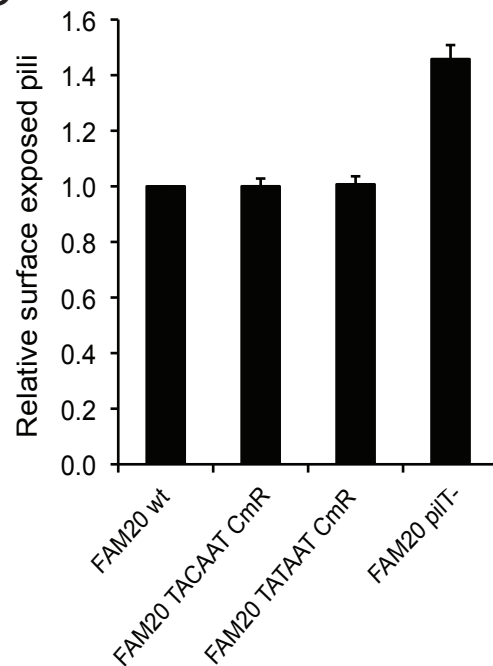

**D**

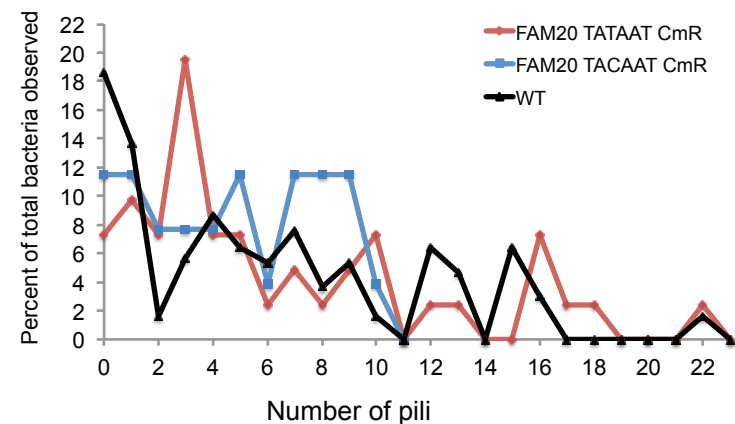

**E**

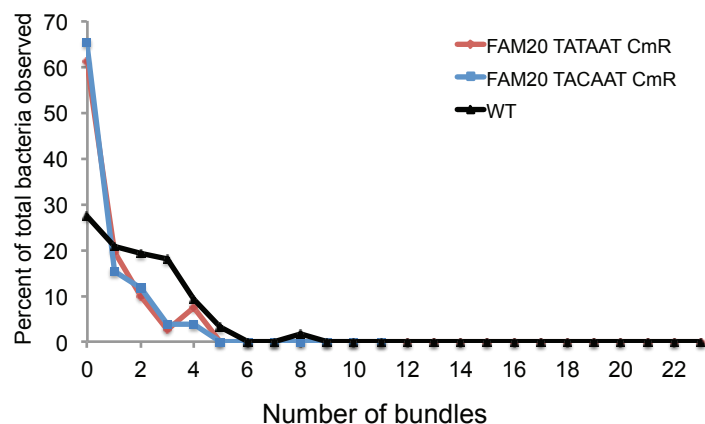

Supplement: Additional file 3: Figure S3. — pilT promoter mutants in N400 (A) and FAM20 (B) were observed using live-cell microscopy, and tracks were analyzed by particle tracking. The data are presented as the average values of at least 40 tracks acquired in two to three independent experiments. The error bars indicate the standard error. The level of surface-exposed pili on meningococcal strains was quantified with whole cell ELISA (C) using an anti-pili antibody that primarily recognizes PilE. The bar chart shows the relative surface-exposed pili levels of the bacterial strains. The absorbance value of FAM20 WT was set to 1.0. The FAM20 ∆pilT strain was included as a hyperpiliated control. The bars represent the mean ± standard deviation from two separate experiments. Quantification of piliation in FAM20 pilT promoter mutants using TEM (D and E) The graphs show the percentage of bacteria observed with x number of single pili (D) or pili bundles (irrespective of bundle width) (E) that appear to emanate from the bacteria. The total number of bacteria observed per strain were: TATAAT CmR n = 41, TACAAT CmR n = 26. Results from FAM20 WT in Figure 5 are included as reference. The mean from two to three independent experiments are shown. [file 12866_2015_424_MOESM3_ESM.pdf]

# Figure S4

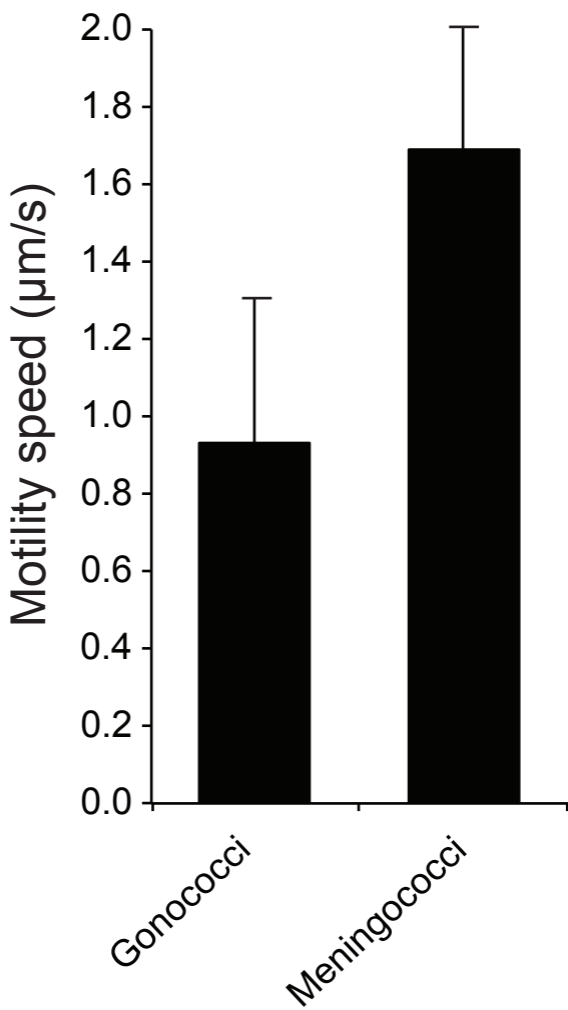

Supplement: Additional file 4: Figure S4. — Average motility of Neisseria strains stained with NHS-ester-based fluorescent dye and observed using live-cell TIRF microscopy. The average speed for gonococcal strains was 0.9 ± 0.4 μm/s (N = 19 tracks), and the average speed for meningococcal strains was 1.7 ± 0.4 μm/s (N = 11 tracks). ± denotes the standard deviation. [file 12866_2015_424_MOESM4_ESM.pdf]

Figure S5

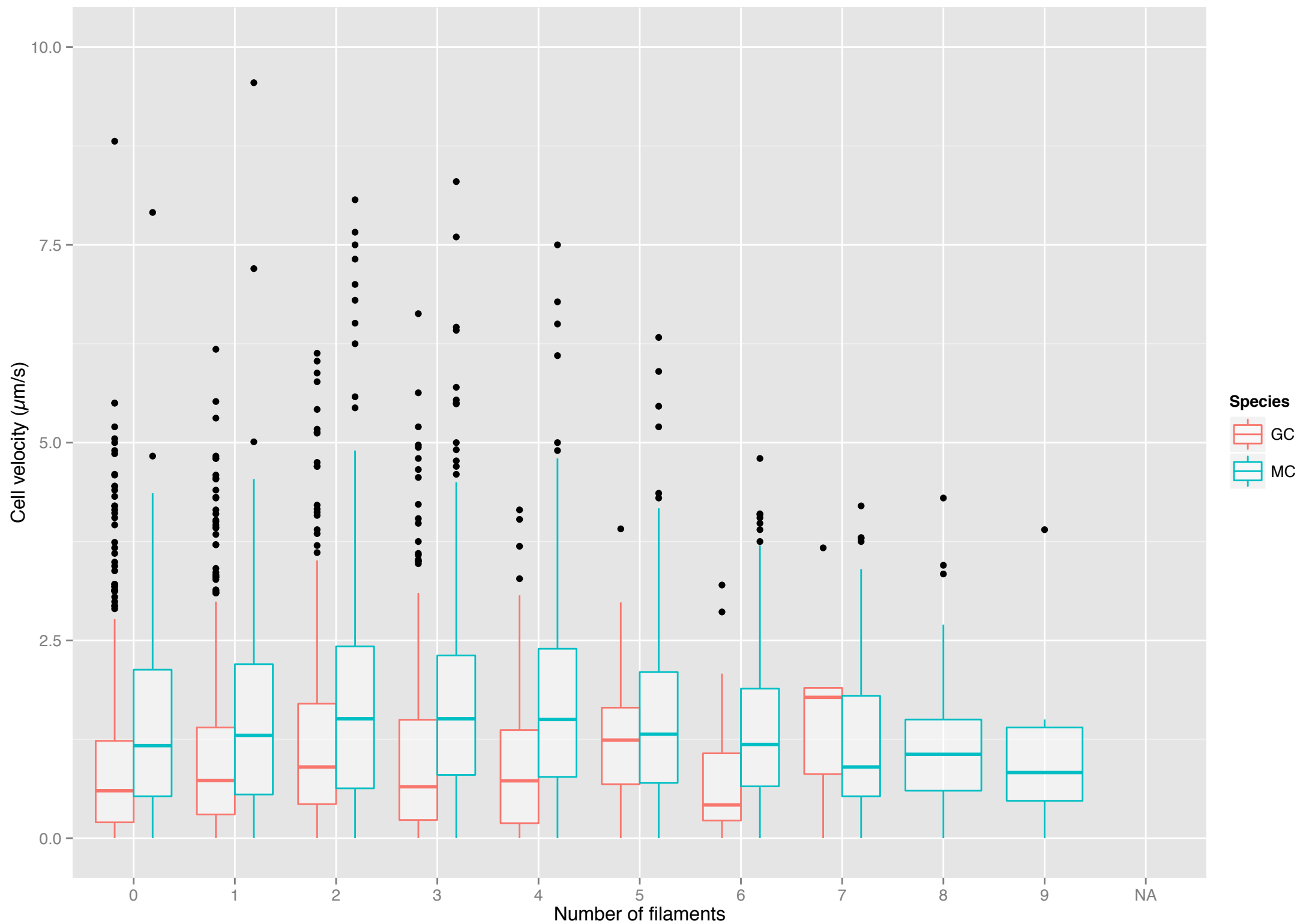

Supplement: Additional file 7: Figure S5. — Relation between the average motility speed and the number of visible filaments in bacteria observed by TIRF. A frame-by-frame analysis of active filaments and bacterial speed in more than 2600 frames for each species was performed. The error bar corresponds to the standard deviation of the velocity. [file 12866_2015_424_MOESM7_ESM.pdf]
