# Supplementary material for: Characterization of motility and piliation in pathogenic Neisseria
Source: BMC Microbiol. 2015 Apr 30;15:92. doi: 10.1186/s12866-015-0424-6 (PMC4449605; doi:10.1186/s12866-015-0424-6)
Supplement: Additional file 2: Figure S2. — Alignment of the pilT-promoter region from all sequenced gonococcal and meningococcal genomes. [file 12866_2015_424_MOESM2_ESM.pdf]

Figure S2

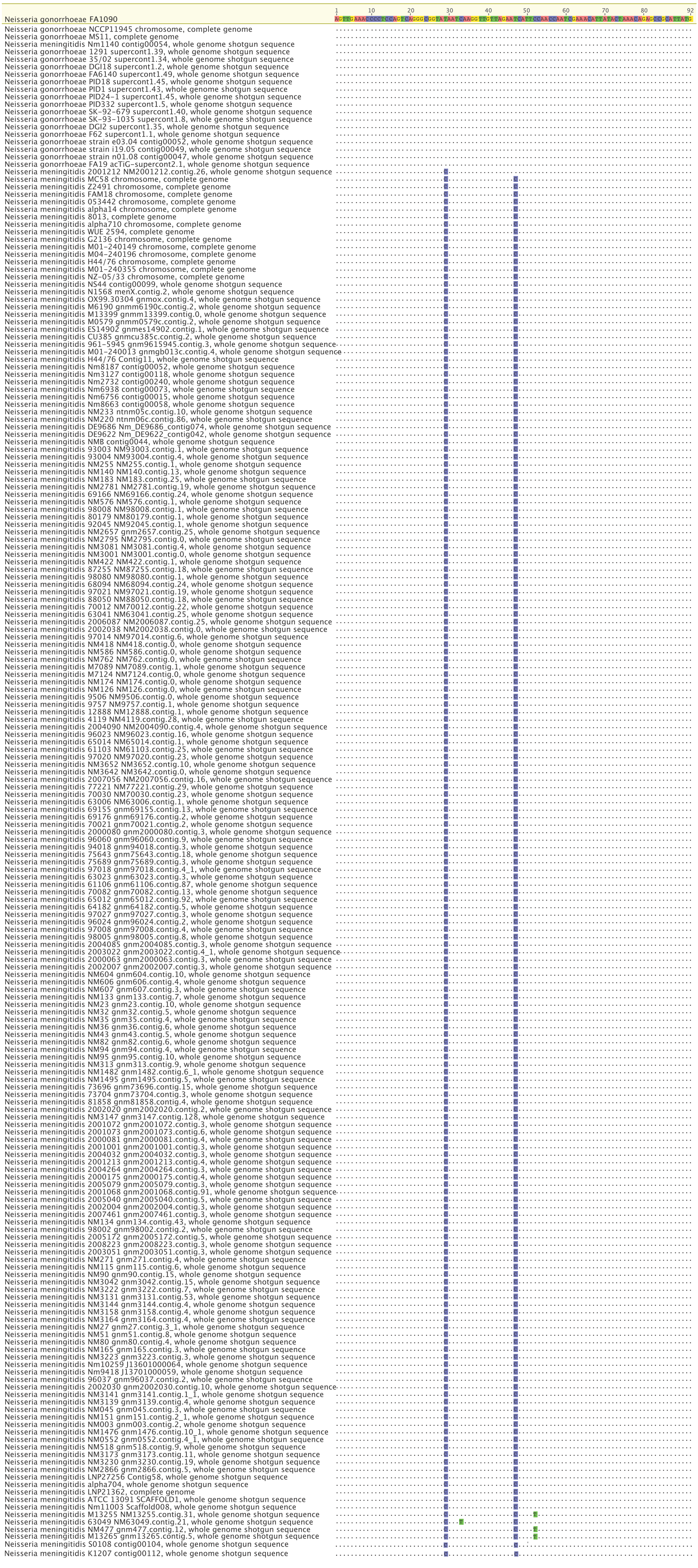
1 10 20 30 40 50 60 70 80 92
Neisseria gonorrhoeae FA1090
Neisseria gonorrhoeae NCCP11945 chromosome, complete genome
Neisseria gonorrhoeae MS11, complete genome
Neisseria meningitidis Nm1140 contig00054, whole genome shotgun sequence
Neisseria gonorrhoeae 1291 supercont1.39, whole genome shotgun sequence
Neisseria gonorrhoeae 35/02 supercont1.34, whole genome shotgun sequence
Neisseria gonorrhoeae DGI18 supercont1.2, whole genome shotgun sequence
Neisseria gonorrhoeae FA6140 supercont1.49, whole genome shotgun sequence
Neisseria gonorrhoeae PID18 supercont1.45, whole genome shotgun sequence
Neisseria gonorrhoeae PID1 supercont1.43, whole genome shotgun sequence
Neisseria gonorrhoeae PID24-1 supercont1.45, whole genome shotgun sequence
Neisseria gonorrhoeae PID332 supercont1.5, whole genome shotgun sequence
Neisseria gonorrhoeae SK-92-679 supercont1.40, whole genome shotgun sequence
Neisseria gonorrhoeae SK-93-1035 supercont1.8, whole genome shotgun sequence
Neisseria gonorrhoeae DGI2 supercont1.35, whole genome shotgun sequence
Neisseria gonorrhoeae F62 supercont1.1, whole genome shotgun sequence
Neisseria gonorrhoeae strain e03.04 contig00052, whole genome shotgun sequence
Neisseria gonorrhoeae strain i19.05 contig00049, whole genome shotgun sequence
Neisseria gonorrhoeae strain n01.08 contig00047, whole genome shotgun sequence
Neisseria gonorrhoeae FA19 acTiG-supercont2.1, whole genome shotgun sequence
Neisseria meningitidis 2001212 NM2001212.contig.26, whole genome shotgun sequence
Neisseria meningitidis MC58 chromosome, complete genome
Neisseria meningitidis Z2491 chromosome, complete genome
Neisseria meningitidis FAM18 chromosome, complete genome
Neisseria meningitidis 053442 chromosome, complete genome
Neisseria meningitidis alpha14 chromosome, complete genome
Neisseria meningitidis 8013, complete genome
Neisseria meningitidis alpha710 chromosome, complete genome
Neisseria meningitidis WUE 2594, complete genome
Neisseria meningitidis G2136 chromosome, complete genome
Neisseria meningitidis M01-240149 chromosome, complete genome
Neisseria meningitidis M04-240196 chromosome, complete genome
Neisseria meningitidis H44/76 chromosome, complete genome
Neisseria meningitidis M01-240355 chromosome, complete genome
Neisseria meningitidis NZ-05/33 chromosome, complete genome
Neisseria meningitidis NS44 contig00099, whole genome shotgun sequence
Neisseria meningitidis N1568 menX.contig.2, whole genome shotgun sequence
Neisseria meningitidis OX99.30304 gnmox.contig.4, whole genome shotgun sequence
Neisseria meningitidis M6190 gnmm6190c.contig.2, whole genome shotgun sequence
Neisseria meningitidis M13399 gnmm13399.contig.0, whole genome shotgun sequence
Neisseria meningitidis M0579 gnmm0579c.contig.2, whole genome shotgun sequence
Neisseria meningitidis ES14902 gnmes14902.contig.1, whole genome shotgun sequence
Neisseria meningitidis CU385 gnmcu385c.contig.2, whole genome shotgun sequence
Neisseria meningitidis 961-5945 gnm9615945.contig.3, whole genome shotgun sequence
Neisseria meningitidis M01-240013 gnmgb013c.contig.4, whole genome shotgun sequence
Neisseria meningitidis H44/76 Contig11, whole genome shotgun sequence
Neisseria meningitidis Nm8187 contig00052, whole genome shotgun sequence
Neisseria meningitidis Nm3127 contig00118, whole genome shotgun sequence
Neisseria meningitidis Nm2732 contig00240, whole genome shotgun sequence
Neisseria meningitidis Nm6938 contig00073, whole genome shotgun sequence
Neisseria meningitidis Nm6756 contig00015, whole genome shotgun sequence
Neisseria meningitidis Nm8663 contig00058, whole genome shotgun sequence
Neisseria meningitidis NM233 ntnm05c.contig.10, whole genome shotgun sequence
Neisseria meningitidis NM220 ntnm06c.contig.86, whole genome shotgun sequence
Neisseria meningitidis DE9686 Nm_DE9686_contig074, whole genome shotgun sequence
Neisseria meningitidis DE9622 Nm_DE9622_contig042, whole genome shotgun sequence
Neisseria meningitidis NMB contig0044, whole genome shotgun sequence
Neisseria meningitidis 93003 NM93003.contig.1, whole genome shotgun sequence
Neisseria meningitidis 93004 NM93004.contig.4, whole genome shotgun sequence
Neisseria meningitidis NM255 NM255.contig.1, whole genome shotgun sequence
Neisseria meningitidis NM140 NM140.contig.13, whole genome shotgun sequence
Neisseria meningitidis NM183 NM183.contig.25, whole genome shotgun sequence
Neisseria meningitidis NM2781 NM2781.contig.19, whole genome shotgun sequence
Neisseria meningitidis 69166 NM69166.contig.24, whole genome shotgun sequence
Neisseria meningitidis NM576 NM576.contig.1, whole genome shotgun sequence
Neisseria meningitidis 98008 NM98008.contig.1, whole genome shotgun sequence
Neisseria meningitidis 80179 NM80179.contig.1, whole genome shotgun sequence
Neisseria meningitidis 92045 NM92045.contig.1, whole genome shotgun sequence
Neisseria meningitidis NM2657 gnm2657.contig.25, whole genome shotgun sequence
Neisseria meningitidis NM2795 NM2795.contig.0, whole genome shotgun sequence
Neisseria meningitidis NM3081 NM3081.contig.4, whole genome shotgun sequence
Neisseria meningitidis NM3001 NM3001.contig.0, whole genome shotgun sequence
Neisseria meningitidis NM422 NM422.contig.1, whole genome shotgun sequence
Neisseria meningitidis 87255 NM87255.contig.18, whole genome shotgun sequence
Neisseria meningitidis 98080 NM98080.contig.1, whole genome shotgun sequence
Neisseria meningitidis 68094 NM68094.contig.24, whole genome shotgun sequence
Neisseria meningitidis 97021 NM97021.contig.19, whole genome shotgun sequence
Neisseria meningitidis 88050 NM88050.contig.18, whole genome shotgun sequence
Neisseria meningitidis 70012 NM70012.contig.22, whole genome shotgun sequence
Neisseria meningitidis 63041 NM63041.contig.25, whole genome shotgun sequence
Neisseria meningitidis 2006087 NM2006087.contig.25, whole genome shotgun sequence
Neisseria meningitidis 2002038 NM2002038.contig.0, whole genome shotgun sequence
Neisseria meningitidis 97014 NM97014.contig.6, whole genome shotgun sequence
Neisseria meningitidis NM418 NM418.contig.0, whole genome shotgun sequence
Neisseria meningitidis NM586 NM586.contig.0, whole genome shotgun sequence
Neisseria meningitidis NM762 NM762.contig.0, whole genome shotgun sequence
Neisseria meningitidis M7089 NM7089.contig.1, whole genome shotgun sequence
Neisseria meningitidis M7124 NM7124.contig.0, whole genome shotgun sequence
Neisseria meningitidis NM174 NM174.contig.0, whole genome shotgun sequence
Neisseria meningitidis NM126 NM126.contig.0, whole genome shotgun sequence
Neisseria meningitidis 9506 NM9506.contig.0, whole genome shotgun sequence
Neisseria meningitidis 9757 NM9757.contig.1, whole genome shotgun sequence
Neisseria meningitidis 12888 NM12888.contig.1, whole genome shotgun sequence
Neisseria meningitidis 4119 NM4119.contig.28, whole genome shotgun sequence
Neisseria meningitidis 2004090 NM2004090.contig.4, whole genome shotgun sequence
Neisseria meningitidis 96023 NM96023.contig.16, whole genome shotgun sequence
Neisseria meningitidis 65014 NM65014.contig.1, whole genome shotgun sequence
Neisseria meningitidis 61103 NM61103.contig.25, whole genome shotgun sequence
Neisseria meningitidis 97020 NM97020.contig.23, whole genome shotgun sequence
Neisseria meningitidis NM3652 NM3652.contig.10, whole genome shotgun sequence
Neisseria meningitidis NM3642 NM3642.contig.0, whole genome shotgun sequence
Neisseria meningitidis 2007056 NM2007056.contig.16, whole genome shotgun sequence
Neisseria meningitidis 77221 NM77221.contig.29, whole genome shotgun sequence
Neisseria meningitidis 70030 NM70030.contig.23, whole genome shotgun sequence
Neisseria meningitidis 63006 NM63006.contig.1, whole genome shotgun sequence
Neisseria meningitidis 69155 gnm69155.contig.13, whole genome shotgun sequence
Neisseria meningitidis 69176 gnm69176.contig.2, whole genome shotgun sequence
Neisseria meningitidis 70021 gnm70021.contig.2, whole genome shotgun sequence
Neisseria meningitidis 2000080 gnm2000080.contig.3, whole genome shotgun sequence
Neisseria meningitidis 96060 gnm96060.contig.9, whole genome shotgun sequence
Neisseria meningitidis 94018 gnm94018.contig.3, whole genome shotgun sequence
Neisseria meningitidis 75643 gnm75643.contig.18, whole genome shotgun sequence
Neisseria meningitidis 75689 gnm75689.contig.3, whole genome shotgun sequence
Neisseria meningitidis 97018 gnm97018.contig.4_1, whole genome shotgun sequence
Neisseria meningitidis 63023 gnm63023.contig.3, whole genome shotgun sequence
Neisseria meningitidis 61106 gnm61106.contig.87, whole genome shotgun sequence
Neisseria meningitidis 70082 gnm70082.contig.13, whole genome shotgun sequence
Neisseria meningitidis 65012 gnm65012.contig.92, whole genome shotgun sequence
Neisseria meningitidis 64182 gnm64182.contig.5, whole genome shotgun sequence
Neisseria meningitidis 97027 gnm97027.contig.3, whole genome shotgun sequence
Neisseria meningitidis 96024 gnm96024.contig.2, whole genome shotgun sequence
Neisseria meningitidis 97008 gnm97008.contig.4, whole genome shotgun sequence
Neisseria meningitidis 98005 gnm98005.contig.8, whole genome shotgun sequence
Neisseria meningitidis 2004085 gnm2004085.contig.3, whole genome shotgun sequence
Neisseria meningitidis 2003022 gnm2003022.contig.4_1, whole genome shotgun sequence
Neisseria meningitidis 2000063 gnm2000063.contig.3, whole genome shotgun sequence
Neisseria meningitidis 2002007 gnm2002007.contig.3, whole genome shotgun sequence
Neisseria meningitidis NM604 gnm604.contig.10, whole genome shotgun sequence
Neisseria meningitidis NM606 gnm606.contig.4, whole genome shotgun sequence
Neisseria meningitidis NM607 gnm607.contig.3, whole genome shotgun sequence
Neisseria meningitidis NM133 gnm133.contig.7, whole genome shotgun sequence
Neisseria meningitidis NM23 gnm23.contig.10, whole genome shotgun sequence
Neisseria meningitidis NM32 gnm32.contig.5, whole genome shotgun sequence
Neisseria meningitidis NM35 gnm35.contig.4, whole genome shotgun sequence
Neisseria meningitidis NM36 gnm36.contig.6, whole genome shotgun sequence
Neisseria meningitidis NM43 gnm43.contig.5, whole genome shotgun sequence
Neisseria meningitidis NM82 gnm82.contig.6, whole genome shotgun sequence
Neisseria meningitidis NM94 gnm94.contig.4, whole genome shotgun sequence
Neisseria meningitidis NM95 gnm95.contig.10, whole genome shotgun sequence
Neisseria meningitidis NM313 gnm313.contig.9, whole genome shotgun sequence
Neisseria meningitidis NM1482 gnm1482.contig.6_1, whole genome shotgun sequence
Neisseria meningitidis NM1495 gnm1495.contig.5, whole genome shotgun sequence
Neisseria meningitidis 73696 gnm73696.contig.15, whole genome shotgun sequence
Neisseria meningitidis 73704 gnm73704.contig.3, whole genome shotgun sequence
Neisseria meningitidis 81858 gnm81858.contig.4, whole genome shotgun sequence
Neisseria meningitidis 2002020 gnm2002020.contig.2, whole genome shotgun sequence
Neisseria meningitidis NM3147 gnm3147.contig.128, whole genome shotgun sequence
Neisseria meningitidis 2001072 gnm2001072.contig.3, whole genome shotgun sequence
Neisseria meningitidis 2001073 gnm2001073.contig.6, whole genome shotgun sequence
Neisseria meningitidis 2000081 gnm2000081.contig.4, whole genome shotgun sequence
Neisseria meningitidis 2001001 gnm2001001.contig.3, whole genome shotgun sequence
Neisseria meningitidis 2004032 gnm2004032.contig.3, whole genome shotgun sequence
Neisseria meningitidis 2001213 gnm2001213.contig.4, whole genome shotgun sequence
Neisseria meningitidis 2004264 gnm2004264.contig.3, whole genome shotgun sequence
Neisseria meningitidis 2000175 gnm2000175.contig.4, whole genome shotgun sequence
Neisseria meningitidis 2005079 gnm2005079.contig.3, whole genome shotgun sequence
Neisseria meningitidis 2001068 gnm2001068.contig.91, whole genome shotgun sequence
Neisseria meningitidis 2005040 gnm2005040.contig.5, whole genome shotgun sequence
Neisseria meningitidis 2002004 gnm2002004.contig.3, whole genome shotgun sequence
Neisseria meningitidis 2007461 gnm2007461.contig.3, whole genome shotgun sequence
Neisseria meningitidis NM134 gnm134.contig.43, whole genome shotgun sequence
Neisseria meningitidis 98002 gnm98002.contig.2, whole genome shotgun sequence
Neisseria meningitidis 2005172 gnm2005172.contig.5, whole genome shotgun sequence
Neisseria meningitidis 2008223 gnm2008223.contig.3, whole genome shotgun sequence
Neisseria meningitidis 2003051 gnm2003051.contig.3, whole genome shotgun sequence
Neisseria meningitidis NM271 gnm271.contig.4, whole genome shotgun sequence
Neisseria meningitidis NM115 gnm115.contig.6, whole genome shotgun sequence
Neisseria meningitidis NM90 gnm90.contig.15, whole genome shotgun sequence
Neisseria meningitidis NM3042 gnm3042.contig.15, whole genome shotgun sequence
Neisseria meningitidis NM3222 gnm3222.contig.7, whole genome shotgun sequence
Neisseria meningitidis NM3131 gnm3131.contig.53, whole genome shotgun sequence
Neisseria meningitidis NM3144 gnm3144.contig.4, whole genome shotgun sequence
Neisseria meningitidis NM3158 gnm3158.contig.4, whole genome shotgun sequence
Neisseria meningitidis NM3164 gnm3164.contig.4, whole genome shotgun sequence
Neisseria meningitidis NM27 gnm27.contig.3_1, whole genome shotgun sequence
Neisseria meningitidis NM51 gnm51.contig.8, whole genome shotgun sequence
Neisseria meningitidis NM80 gnm80.contig.4, whole genome shotgun sequence
Neisseria meningitidis NM165 gnm165.contig.3, whole genome shotgun sequence
Neisseria meningitidis NM3223 gnm3223.contig.3, whole genome shotgun sequence
Neisseria meningitidis Nm10259 J13601000064, whole genome shotgun sequence
Neisseria meningitidis Nm9418 J13701000059, whole genome shotgun sequence
Neisseria meningitidis 96037 gnm96037.contig.2, whole genome shotgun sequence
Neisseria meningitidis 2002030 gnm2002030.contig.10, whole genome shotgun sequence
Neisseria meningitidis NM3141 gnm3141.contig.1_1, whole genome shotgun sequence
Neisseria meningitidis NM3139 gnm3139.contig.4, whole genome shotgun sequence
Neisseria meningitidis NM045 gnm045.contig.3, whole genome shotgun sequence
Neisseria meningitidis NM151 gnm151.contig.2_1, whole genome shotgun sequence
Neisseria meningitidis NM003 gnm003.contig.2, whole genome shotgun sequence
Neisseria meningitidis NM1476 gnm1476.contig.10_1, whole genome shotgun sequence
Neisseria meningitidis NM0552 gnm0552.contig.4_1, whole genome shotgun sequence
Neisseria meningitidis NM518 gnm518.contig.9, whole genome shotgun sequence
Neisseria meningitidis NM3173 gnm3173.contig.11, whole genome shotgun sequence
Neisseria meningitidis NM3230 gnm3230.contig.19, whole genome shotgun sequence
Neisseria meningitidis NM2866 gnm2866.contig.5, whole genome shotgun sequence
Neisseria meningitidis LNP27256 Contig58, whole genome shotgun sequence
Neisseria meningitidis alpha704, whole genome shotgun sequence
Neisseria meningitidis LNP21362, complete genome
Neisseria meningitidis ATCC 13091 SCAFFOLD1, whole genome shotgun sequence
Neisseria meningitidis Nm11003 Scaffold008, whole genome shotgun sequence
Neisseria meningitidis M13255 NM13255.contig.31, whole genome shotgun sequence
Neisseria meningitidis 63049 NM63049.contig.21, whole genome shotgun sequence
Neisseria meningitidis NM477 gnm477.contig.12, whole genome shotgun sequence
Neisseria meningitidis M13265 gnm13265.contig.5, whole genome shotgun sequence
Neisseria meningitidis S0108 contig00104, whole genome shotgun sequence
Neisseria meningitidis K1207 contig00112, whole genome shotgun sequence
